# Ground Reaction Forces Are Predicted with Functional and Clinical Tests in Healthy Collegiate Students

**DOI:** 10.3390/jcm9092907

**Published:** 2020-09-09

**Authors:** Paul A. Cacolice, Christopher R. Carcia, Jason S. Scibek, Amy L. Phelps

**Affiliations:** 1 Department of Movement Science, Sport, and Leisure Studies, Westfield State University, Westfield, MA 01086, USA; 2 Department of Kinesiology, Colorado Mesa University, Grand Junction, CO 81501, USA; ccarcia@coloradomesa.edu or; 3 Rangos School of Health Science, Duquesne University, Pittsburgh, PA 15282, USA; scibekj@duq.edu; 4 Palumbo Donahue School of Business, Duquesne University, Pittsburgh, PA 15282, USA; phelpsa@duq.edu

**Keywords:** anterior cruciate ligament, functional test, injury prediction

## Abstract

Increased vertical and posterior ground reaction forces (GRFs) are associated with anterior cruciate ligament (ACL) injury. If a practical means to predict these forces existed, ACL injury risk could be attenuated. Forty-two active college-age individuals (21 females, 20.66 ± 1.46 y, 70.70 ± 2.36 cm, 82.20 ± 7.60 kg; 21 males, 21.57 ± 1.28 y, 65.52 ± 1.87 cm, 64.19 ± 9.05 kg) participated in this controlled laboratory study. GRFs were ascertained by having the subjects perform a unilateral landing task onto a force plate. Several clinical measures (Fat Free Mass (FFM), dorsiflexion passive range of motion (DPROM), isometric peak force of the lateral hip rotators, knee flexor/extensor peak force ratio (H:Q), the completion of the overhead deep squat), two functional tests (Margaria–Kalamen, Single Leg Triple Hop (SLTH)), and sex served as the predictor variables. Regression models to predict the GRFs normalized to the FFM (nGRFz, nGRFy) were generated. nGRFz was best predicted with a linear regression equation that included SLTH and DPROM (adjusted *R*^2^ = 0.274; *p* = 0.001). nGRFy was best predicted with a linear regression equation that included H:Q, FFM, and DPROM (adjusted *R*^2^ = 0.476; *p* < 0.001). Simple clinical measures and functional tests explain a small to moderate amount of the variance associated with the FFM normalized vertical and posterior GRFs in active college-age individuals.

## 1. Introduction

Anterior cruciate ligament (ACL) injuries are common, costly, and debilitating. It has been estimated that 80,000 to 250,000 ACL injuries occur in the US each year [1,2,3], with an estimated total annual cost to society of between $8 and $18 billion [4]. ACL injury increases the likelihood of re-injury [5,6] and the risk of developing knee osteoarthritis [5]. Furthermore, individuals often face psycho-sociological challenges during their time away from regular activity [7,8]. With such short- and long-term effects of ACL injury, health care professionals should strive to prevent rather than treat these devastating injuries.

To prevent an injury, one must first acquire an understanding of the causal risk factor [9]. More than 30 years of research has shown, however, that there are multiple ACL injury risk factors [1,10,11,12]. Previous work has identified two general areas of risk: intrinsic and extrinsic factors [1,10,11,12]. Intrinsic factors have been classified into anatomic risk factors and hormonal risk factors [1,10]. Intrinsic risk factors originate within the body of the individual and are recognized as under less control [13]. Extrinsic factors originate outside of the human body [1]. These factors have been classified into environmental and biomechanical risks [1,10]. Unlike intrinsic factors, extrinsic factors are recognized as being under some degree of control and hence are modifiable [13]. The utilization of extrinsic factors then should provide a more direct pathway to injury prevention.

Of the recognized extrinsic risk factors, lower extremity kinematics is often examined as a means of identifying ACL injury risk, but is faced with challenges to injury predictive ability [14]. An identified risk factor that has faced less challenges to predictive ability is the inability to dissipate energy from landing or rapidly changing directions. Indeed, previous work has specifically noted that undissipated landing energy can prospectively predict ACL injury incidence [15]. Undissipated landing energy is measured by assessing the ground reaction force (GRF) [15,16,17]. Unfortunately, the quantification of GRFs requires specialized, costly equipment and trained personnel. If clinical measures and functional tests could accurately predict the GRF, then such a strategy would provide a practical ACL injury risk identification strategy. Therefore, the purpose of this study was to determine if practical, clinical measures and functional tests were capable of predicting the vertical and posterior ground reaction forces in an active collegiate population.

## 2. Experimental Section

To investigate the ability of clinical measures and functional tests to predict ground reaction forces, we utilized an observational, descriptive design and received university ethical approval (Duquesne University, Institutional Review Board, Protocol 2015-03-8). Procedures were performed in a controlled, university laboratory setting during a single 75 min session for each participant. Five clinical measures, two functional tests, and sex served as the eight independent variables. The five clinical measures consisted of the determination of Fat Free Mass (FFM), the ankle dorsiflexion passive range of motion (DPROM), the completion of the overhead deep squat (ODS), the hip lateral rotator muscles peak force (HipLR), and the hamstring to quadriceps peak force ratio (H:Q). The two functional tests were the Margaria–Kalamen test (MK) and the Single Leg Triple Hop test (SLTH). These specific measures and tests were selected as they have each previously been utilized to explore predictive ability for ACL injury risk [18,19]. The Fat Free Mass normalized vertical (nGRFz) and posterior ground reaction forces (nGRFy) ascertained during a single limb drop landing served as the two dependent variables. Normalization to FFM was conducted to allow for comparisons between participants of different body masses and sizes [20,21].

The initial inclusion criteria required the participants to be active, healthy collegiate-aged individuals between the ages of 18 and 24. To investigate any sex differences with the dependent or independent variables, equal numbers of females and males were tested. All the participants self-reported that they participated in physical activity on an average of three or more days per week in the past six months. Participants were excluded from participation in the study if: (1) they had any lower extremity (LE) joint surgery, (2) they self-reported any neurological or neuromotor condition which may affect muscle strength or coordination, (3) they utilized crutches for any LE injury over the last six months, (4) they participated in any formal rehabilitation program for a LE injury in the past six months, (5) they could not perform any of the required testing procedures in the investigation, (6) they scored less than 71% on the Lower Extremity Functional Scale, or (7) they scored greater than 6 on the Beighton hypermobility scale.

As FFM equations assume the body fluids are within specific parameters [22], any factor affecting the normal body fluid levels would limit an individual’s immediate study participation. All the testing was performed in a temperature and humidity-controlled environment of the university’s motion analysis laboratory to reduce the potential for heat stress during data collection. Individuals were instructed during scheduling to avoid alcohol, caffeine, diuretics, or excessive amounts of fluid 12 h prior to the testing session [22]. To reduce the error associated with fluid retention in females, testing was not scheduled seven days before or after the self-reported start of menstruation [23]. In addition, the individuals were instructed not to exercise within six hours of study participation, as exercise causes fluid shifts to the skin and peripheral muscles [24]. The participants were also instructed to refrain from large meals prior to the testing session, as has been established practice with skinfold measurements [24]. Altogether, these pre-testing standards to establish consistent body fluid levels for FFM measures would likely be more stringent than could be expected from pre-participation preparation in young adult amateur athletes. As the tested individuals represent the population at greater risk for ACL injury, we felt that placing even greater standards on the individuals than they might face in a realistic scenario could result in a violation of our testing sample.

Upon obtaining written informed consent, the individuals completed the Lower Extremity Function Scale (LEFS) to assess LE function [25,26,27]. Individuals that scored less than 71/80, indicating LE functional limitations, were excused from further participation. This value was identified as the point at which there is a minimal detectable change for loss of lower extremity function utilizing the LEFS [25]. Following the LEFS, the individual was prompted by the lead investigator to perform the tests of the Beighton Hypermobility Scale [28]. A score of 4–6 in young adults indicates hypermobility [29]. Individuals that scored greater than six out of the nine possible points, indicating excessive risk of injury for field and court-based sport athletes [30], were excused from further participation.

**Figure 1:**
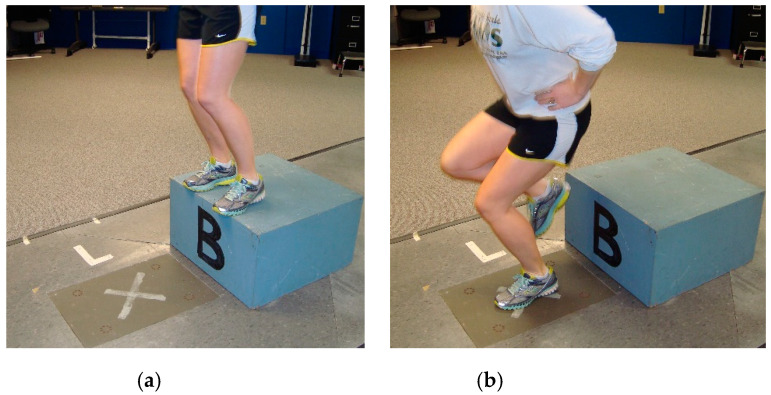
Participant body positioning for the drop-land: (**a**) forward lean off of platform; (**b**) single limb landing.

The height and mass of the participant were determined with the utilization of a scientific grade medical beam balance scale and stadiometer (Jarden Corporation, Rye, NY, USA). The participant then stood on a 35 cm-high wooden platform with their toes on the leading edge of the platform and hands on their iliac crests (Figure 1). While maintaining their hands on their hips, the participant leaned forward and off the platform, landing on one foot with no additional secondary hop. The foot chosen for landing by the participant on two out of three trials was defined as the dominant LE [31]. After determination of the dominant LE, the quantification of independent and dependent measures commenced. The test order was specified to prevent bias from fluid redistribution due to exercise [22].

### 2.1. Test 1—Fat Free Mass (FFM) Assessment

The literature that suggests the strength of multiple LE muscles working together affects force dissipation and thus prevents injury to the passive restraints at the knee. Unfortunately, the assessment of each muscle involved would require considerable time. The utilization of a method to assess cumulative LE strength should provide an effective estimate of potential force absorption.

The amount of output a muscle may generate is related to the muscle cross-sectional area (*r =* 0.51–0.92) [32,33,34] and the total mass of the muscle unit [35]. Yet, simply determining the individual’s overall mass could prove problematic if an individual has a greater mass due to greater adiposity. Likewise, the percentage of body composition alone limits information as to the mass of the musculature. The accurate differentiation of increased muscle mass versus increased overall mass is required.

A practical measure of greater combined muscular cross-section throughout the body could occur with an assessment of an individual’s Fat Free Mass (FFM) [22]. Many FFM determination methods utilize a limited number of readily assessable skinfold sites. The individual’s muscle mass could in theory provide a practical assessment of an individual’s ability to dissipate landing force. Additionally, females tend to have higher percent of body fat and therefore lower FFM levels compared to males of the same body mass. If the above rationale holds, females would have less ability to dissipate GRFs than males of the same body mass due to differences in FFM.

Skinfold measurements were taken using the Jackson–Pollock method. For females, skinfolds are taken at the triceps, the supra-iliac, and at the thigh sites. For males, the skinfold sites are the chest, the abdomen adjacent to the umbilicus, and the thigh. To minimize inter-tester error, the primary investigator was the sole individual taking the skinfold (SKF) measurements [36,37]. In addition, the calipers (Lange, Beta Technologies, Santa Cruz, CA, USA) utilized exclusively in the study were calibrated for measured distance prior to the start of the study [22,38].

The participant’s skin was dry and free from lotion [22]. Sites for each skinfold were taken on the right side of the body, independent of the participant’s side dominance [22]. All the SKF sites were identified, marked, and taken at each site in a rotating order [22,39]. After the first reading for all sites, the pattern was repeated for a total of three readings [22] with the mean of the three values at each site recorded. If any reading varied more than 10%, additional readings were taken [22].

The sum of the three skinfolds Σ3SKF) was calculated for each individual, and the FFM was calculated using standard equations [22]. The primary investigator’s intra-rater reliability of SKF was established prior to this study and was deemed “clinically reliable” (ICC(_3,k_) = 0.996, SEM = 2.826 mm, *p* < 0.001) [40].

### 2.2. Test 2—Ankle Dorsiflexion Passive Range of Motion (DPROM)

The participant assumed a supine position, with their upper body propped onto their elbows and their ankle extended over the end of a padded, treatment plinth. The participant’s knee was placed in full extension. In this position, the participant’s ankle was passively moved into dorsiflexion to end range and measured with a standard goniometer (JA Preston, Jackson, MI, USA) [41]. The mean of three trials was utilized in accordance with the existing literature [42]. The primary investigator’s intra-rater reliability of DPROM was established prior to this study and was deemed “clinically reliable” [40] (ICC(_3,k_) = 0.937, SEM = 1.669°, *p* < 0.001) and comparable to findings in the literature [43].

### 2.3. Test 3—Two-Legged, Overhead Deep Squat (ODS)

For the completion of this activity, the participant was first asked to stand upright with their feet shoulder width apart and facing anteriorly. The participant was given a polyvinyl chloride 1” diameter pipe (North American Pipe Corporation, Houston, TX, USA) and asked to grab one end with each hand as the pipe rested on top of their head. The participant’s hands were moved along the length of the pipe until the participant had a 90° shoulder abduction and 90° elbow flexion. After the confirmation of proper grip width by the lead investigator, the participant was asked to elevate and keep the pipe overhead to main the parallel relationship of the torso to the leg (shank). Once overhead, the participant was asked to squat down as low as possible, moving the lower extremities primarily in the sagittal plane while keeping their heels on the floor. The participant was asked to continue the squat until their thighs were below parallel with the floor and with their knees directly over their toes. If the participant was able to complete the movement as described, for the purposes of this investigation the subject received a “1”. If the participant was unable to complete the activity as specifically detailed above for any reason, they received a “0”.

### 2.4. Test 4—Landing Kinetics

The participants performed a ten-minute warm-up on a stationary bike (Monarch Ergo-Medic Monarch, Vansbro, Sweden) at a self-selected pace. A self-selected warm-up has been established in the literature prior to landing analysis when any warm-up is utilized [44,45]. The participant then stood on the 35 cm-high wooden platform, with their toes on the leading edge of the platform and hands on their iliac crests. As during the assessment of LE dominance, the participant leaned forward and off of the platform, maintaining their hands on their iliac crests, landing on one foot with no additional secondary hop. For these landings, the participant performed a total of five single-limb drop landings with the dominant LE onto a six-degree of freedom forceplate (Bertec Corporation, Columbus, OH, USA). An inability to land in the middle of the forceplate, keep their hands on their iliac crests, or land without a secondary hop resulted in the negation of that trial. The forceplate was set with a threshold of 13.345 N and recorded 0.100 s before and 0.900 s after the threshold force was met. After five successful trials, the participants were given a three-minute rest before proceeding with the rest of the study.

The participants then performed the following tests in a counter-balanced order. A rest period of three minutes between each of these tests was provided to minimize the effects of fatigue on performance.

### 2.5. Test 5—Lower Extremity Power Measured with the Margaria–Kalamen Test (MK)

The MK has been a standard of fitness testing since 1964 and has a “good” test-retest reliability (ICC = 0.73) [46]. In our investigation, each participant first stood with their toes on a marked line, six meters from and level with the first step of an 11-step staircase that had a rise of 16.6 cm per step. A pressure-switch-triggered digital timer (Lafayette Instrument, Lafayette, IN, USA) was placed on the third and ninth step. On the investigator’s signal, the participant ran from the starting mark as fast as possible and bound up the stairway taking the steps three at a time (third, sixth, ninth). The timer started recording when the participant contacted the third step and stopped recording when the participant contacted the ninth step. To assure proper foot placement, the third, sixth, and ninth steps were marked with small brightly colored cones (Lakeside Plastics, Oshkosh, WI, USA). The participant completed three trials with a 20 s rest between each trial, as is established practice in the research and clinical literature. The best performance time (t) was used to calculate the participant’s peak power (*p* = (Mass × Vertical distance between 9th and 3rd step) × 9.8 ÷ t) [47].

### 2.6. Test 6—Test E—Single-Limb Triple Hop (SLTH)

The test-retest reliability of the SLTH has been established (ICC = 0.80–0.97) [48,49] and previously utilized in assessing ACL function in collegiate-aged females [19]. To perform this test, each participant placed the heel of their dominant LE at the leading edge of a marked line while keeping their hands on their iliac crests throughout the activity. They then performed three sequential, dominant LE hops while achieving the greatest horizontal distance possible. The participants were encouraged to spend the least amount of time possible in contact with the ground until landing the third hop. Four practice trials were performed prior to measured trials. The first practice trial was performed at 50% effort, the second at 75% effort, and the third and fourth trial at maximal effort. The individual performed three maximal trials with a 15 s recovery between trials. This procedure, including recovery time, is consistent with previously established practice [50]. Upon completion of each test trial, the investigator measured the horizontal distance hopped from the starting line to the heel of the third landing with a standard tape measure (American Guidance Service, Inc., Circle Pines, MN, USA). The mean of the three measured trials was utilized for data analysis [19,50,51].

### 2.7. Test 7—Hamstring to Quadriceps Ratio of Isometric Peak Force Contraction (H:Q)

To calculate the H:Q, the ratio of Knee Flexor isometric peak force (KF) was divided by Knee Extensor isometric peak force (KE).

#### 2.7.1. Knee Flexor Isometric Peak Force Contraction (KF)

The participant was seated with their upper body perpendicular to and with their knee over the end of a padded, treatment plinth [52]. The participant’s arms were crossed over the chest and the hands were kept open. The participant was instructed to keep the torso upright and not lean backward or forward. With one hand, the investigator held the handheld dynamometer (HHD) (Lafayette Manual Muscle Test System, Model 01165, Lafayette Instruments, Lafayette, IN, USA) on the posterior side of the dominant LE just proximal to the level of the malleoli. The investigator’s other hand was placed on the anterior/distal aspect of the thigh. The participant placed the knee in a position of 90° flexion [53,54]. The participant was instructed to “pull as hard as you can to bend the knee”. The investigator provided force in an effort to prevent the movement of the dominant LE. The tested activity period was held for five seconds. The participant was given a 30 s recovery period between each test bout. This recovery time was established to provide an adequate work:recovery ratio, thus providing investigators expectations of complete recovery for an activity [55]. Three repetitions were completed, and the mean peak KF force recorded was calculated.

#### 2.7.2. Knee Extensor Isometric Peak Force Contraction (KE)

The participant positioning for KE was identical to that described for KF. The HHD was, however, positioned on the anterior side of the dominant LE, just proximal to the level of the malleoli. The participant was instructed to “push as hard as you can to straighten the knee”. The investigator provided force to prevent movement of the dominant LE. The tested activity period was held for five seconds. The participant was given a 30 s recovery period between each test bout with a rationale as noted above [55]. Three repetitions were completed, and the mean peak KE force recorded was calculated.

The primary investigator’s intra-rater reliability with a HHD was established prior to this study for knee flexion (ICC_(3,k)_ = 0.864, SEM = 23.232 N, *p* = 0.003) and extension (ICC_(3,k)_ = 0.870, SEM = 26.597 N, *p* = 0.003) for knee extension. The reliability coefficients were comparable to reported values in the literature [53] and deemed “good” and “clinically reliable” [40].

### 2.8. Test 8—Hip Lateral Rotator Isometric Peak Force Contraction (HipLR)

As with the KF/KE measures, the participant sat on the edge of a plinth. Arms were crossed over the chest, hands kept open, and the hips and knees were flexed to 90°. To record the measure, the HHD was held against the participant by the investigator at a point just proximal to the medial malleolus. The investigator’s other hand applied counter-pressure over the lateral aspect of the distal thigh, just proximal to the knee [52]. The participant was instructed to “push as hard as you can to move your ankle inward”. The investigator provided force to prevent lateral (external) rotation of the hip. The tested activity was held for five seconds. Three repetitions were completed and the mean peak force recorded was calculated. The primary investigator’s intra-rater reliability of HipLR was established prior to this study and was deemed “clinically reliable” (ICC_(3,k)_ = 0.977, SEM = 9.419 N, *p* < 0.001) [40].

#### 2.8.1. Statistical Analyses

The three-step calculation process to determine the FFM was performed and recorded for each participant. The landing data for GRFz and GRFy were signal-averaged and harvested with the data analysis software (Datapac 2000, Run Technologies, Laguna Hills, CA, USA). The peak amplitude for GRFz and GRFy were subsequently normalized for the individual’s FFM (nGRFz and nGRFy, respectively).

Data for all the dependent and independent variables along with height, mass, age, and numbers of days participating in physical exercise each week were entered into a statistical software package (SPSS-22, IBM; Armonk, NY, USA). Descriptive statistics were compiled for all the dependent and independent variables, along with demographic data. Two separate step-wise linear regression models using the “Enter” method were calculated. One model predicted nGRFz, while the second model predicted nGRFy. Both the models used the results from the independent variables (clinical and functional tests) as the predictors. The coefficient of determination (*r*^2^) and analysis of the variance of regression from each model were generated along with an analysis of the residuals and outliers. Further, the inter-correlations amongst and between the independent and dependent variables were assessed. The alpha levels for all the analyses were set a priori at *p* ≤ 0.05.

#### 2.8.2. Power Analysis

An a-priori power analysis was performed utilizing available statistical software (G*Power v3.1.9.2, Düsseldorf, Germany). The effect sizes from existing published data for our selected independent variables on GRFy and GRFz were harvested when available [18,42,56]. The smallest reported correlations for between our independent variables and our two dependent variables were 0.829 (GRFy) and 0.998 (GRFz). The sample size was calculated utilizing the “Linear multiple regression: Fixed model, *r*^2^ deviation from zero” option in the “f^2^ Test” menu. Using the smaller effect size of the two (0.829) and “eight” as the number of predictors, G*Power returned a calculated sample size of 27 to achieve a desired level of power of 0.80. To account for any error in estimating effect size, a sample of 42 was recruited for participation in the current study.

## 3. Results Section

### 3.1. Demographic Analysis

Forty-four individuals were recruited for the study. Two individuals were excluded due to previous injury that might affect the sample assumptions. Of these two individuals, one had been in a previously unreported rehabilitation program for an LE muscular strain. The other individual had suffered a concussion which precluded her participation due to the elevated risk of lower extremity injury [57]. Thus, forty-two individuals were then utilized in the final data analyses (21 females and 21 males). Descriptive statistics for the participants are reported in Table 1.

### 3.2. Descriptive Analysis

The means and standard deviations for all the independent and dependent variables are reported in Table 2. The mean time to peak GRFz occurred at 0.060 ± 0.014 s, while the time to peak GRFy occurred at 0.035 ± 0.031 s.

### 3.3. Correlation and Chi-Squared Analysis

A correlation matrix displaying the correlations amongst and between the continuous independent variables for nGRFz is presented in Table 3. A correlation matrix displaying the correlations amongst and between the continuous independent variables for nGRFy is presented in Table 4. The point bi-serial correlations between the two dichotomous variables (sex, ODS) and continuous measures are detailed in Table 5. A chi-squared analysis of the dichotomous independent variable (ODS) to sex is explored in Table 6.

### 3.4. Regression Analysis with the Examination of Residuals and Outliers

A stepwise linear regression analysis of the eight-predictor variable model for nGRFz resulted in a statistically significant model (*p* = 0.048). Further evaluation, however, indicated that the most parsimonious model occurred when utilizing only SLTH and DPROM as predictor (independent) variables (Adjusted *R*^2^ = 0.274; *p* = 0.001) (Table 7). The nGRFz model is expressed with the equation:

nGRFz = 7.868 − 0.006(SLTH) − 0.055(DPROM).(1)

Stepwise linear regression analysis of an eight-predictor variable model for nGRFy also resulted in a statistically significant model (*p* = 0.001). Further evaluation provided that the most parsimonious model occurred when utilizing only H:Q, FFM, and then DPROM as predictor (independent) variables. The resulting model had an adjusted *R*^2^ = 0.476 and was significant *p* < 0.001 (Table 8). The remaining predictor (independent) variables did not significantly contribute to the prediction of nGRFy. The nGRFy model is expressed with the equation:

nGRFy = −4.394 − 2.579(H:Q) + 0.041(FFM) + 0.041(DPROM).(2)

### 3.5. Post Hoc Power Analysis

To determine the post hoc power for each linear regression model, a Cohen’s *f*^2^ value was first calculated from the regression models’ *r*^2^ value utilizing the equation *f*^2^ = *r*^2^/(1 − *r*^2^). The resulting effect size was small to medium [40] (*p* 649) for nGRFz (Cohen’s *f*^2^ = 0.377) and large [40] (p649) for nGRFy (Cohen’s *f*^2^ = 0.908). The resultant effect sizes, sample size, and alpha error size were entered into a commercially available power analysis software package (G*Power, v 3.1.2, Düsseldorf, Germany). The software generated a post hoc power of 0.803 for the nGRFz regression model and 0.818 for the nGRFy regression model.

## 4. Discussion

The aim of this study was to generate predictive GRF models from clinical and functional tests in a healthy and active college-age population. We hypothesized that such practical tests would predict a significant amount of the variance in the regression models based upon our pilot study work (MK, SLTH), previous literature (DPROM [42,58], HipLR [56], and sex [17,59,60,61,62,63,64,65,66,67,68]), and theory (H:Q, FFM, and ODS). Both nGRFz and nGRFy could be significantly predicted, but this most parsimoniously occurred with the results of select and not all tested predictor variables. The nGRFz model was able to explain 27% of the variance. In the nGRFy model, 48% of the equation variance was explained by the select predictor variables.

### 4.1. Examination of the nGRFz Model

The linear regression analysis specified that the use of all eight independent variables returned a statistically significant model (*p* = 0.048). A step-wise analysis, however, denoted that the use of SLTH and DPROM generated a significant model (*p* = 0.001). The addition of any of the other six independent variables did not significantly add to the model’s predictive ability.

The distance achieved in the SLTH was inversely correlated to nGRFz (*r* = −0.399, *p* = 0.009), as was DPROM (*r =* −0.336, *p =* 0.030). As the SLTH increased in distance, the vertical landing energy was better dissipated. Additionally, as ankle passive dorsiflexion range of motion increased, the vertical landing energy was better dissipated. The addition of DPROM did add to the overall predictive ability (*r* = 0.138 to *r =* 0.274) and improve the level of model statistical significance (*p =* 0.009 to *p =* 0.001). When utilized in the regression analysis, MK and H:Q did not significantly add to the robustness of the model (*p =* 0.409 and *p* = 0.907, respectively).

SLTH requires three successive cycles comprised of landing energy dissipation and take-off force generation all in an exceptionally short time window. In fact, the more rapid and efficient the transition from landing to take off, the greater the SLTH distance achieved [19]. We know from previous work that the greater the rate of energy dissipation in the first 0.1 s, the lesser the peak nGRFz [69]. Our findings of an inverse relationship between SLTH and nGRFz indeed showed that the greater the distance the individual is able to cover with the SLTH, the greater their ability to disperse vertical landing energy with a dominant LE landing.

Hamilton et al. [70] showed that SLTH distance was a predictor of the hamstring peak torque at 60°/s (*r =* 0.753, *p* < 0.01) and 180 °/s (*r =* 0.745, *p* < 0.01). SLTH was also a predictor of the quadriceps peak torque at 60°/s (*r =* 0.700, *p* < 0.01) and 180°/s (*r =* 0.767, *p* < 0.01). Additionally, the vertical jump height (*r =* 0.834, *p* < 0.01) was also correlated with SLTH. Each of these measures examines the muscular output at the knee. The high degree of correlation between SLTH and other measures of muscular output may be why only one variable measuring muscular output in the current investigation (SLTH) provided significant value to the nGRFz regression.

The participants in our investigation displayed a greater ability to disperse vertical landing energy when they displayed a greater DPROM. Fong et al. reported that this same measure of ankle dorsiflexion also predicted the vertical landing force [42]. The authors of that study did utilize a similarly described sample of active, college-age student volunteers as were used in our study. However, the authors determined LE dominance as the preferred LE to maximally kick a ball in contrast to our methodology. Although it may be possible that there are different vertical landing energy dissipation characteristics between the preferred LE for landing and for maximally kicking a ball, our findings did not suggest this.

There was a significant correlation between ODS and DPROM (*r*_pb_ = 0.473, *p =* 0.002) in the current study. There was only significance at the *p* ≤ 0.010 level between ODS and nGRFz. (*r =* −0.267, *p* = 0.087). Our findings support that ankle dorsiflexion taken in a non-fixed foot position (DPROM) provided more information to predict nGRFz than combined LE joint motion with a fixed foot (ODS). It follows that, since ODS utilized ankle dorsiflexion range of motion as one of several components, the additional information provided by the ODS was not beneficial to the predictive ability of the model. The ODS describes active LE joint ranges of motion with gravity. The most likely factors contrasting ODS to DPROM involve motion at the knee, hip, and thoracolumbar joints and the muscular control against gravity.

None of the additional independent variables were able to significantly add to the predictive ability of the model beyond the use of SLTH and DPROM. As SLTH and MK were significantly correlated (*r =* 0.752, *p* < 0.001), the information that each variable provided to the regression overlapped. Individually, FFM (*r =* −0.258, *p =* 0.098) and HipLR (*r =* −0.186, *p =* 0.238) were not significantly correlated to nGRFz. Our findings were contradictory to the rationale we presented for the selection of these variables. SLTH was significantly correlated to FFM (*r =* 0.694, *p* < 0.001), HipLR (*r =* 0.511, *p =* 0.001), and H:Q (*r =* −0.407, *p =* 0.008). As such, the information provided by FFM, MK, and H:Q to the model was better addressed by SLTH.

### 4.2. Examination of the nGRFy Model

The linear regression modeling confirmed that the use of all eight variables returned a statistically significant model (*r*^2^ = 0.476, *p =* 0.001). From the step-wise analysis though, the regression using only H:Q, FFM, and DPROM generated the most economical model (*r*^2^ = 0.479, *p* < 0.001). The addition of the other five predictor variables did not significantly add to the predictive ability of the model.

Taken together, as a participant in our study displayed greater peak hamstring force relative to their peak quadriceps force, the individual landed with a greater posterior GRF. An increased H:Q peak force ratio, then, should decrease the posterior GRF by preventing the anterior translation of the tibia with a co-contraction. This inverse relationship between the H:Q and nGRFy provides support for the protective mechanism of the quadriceps in closed chain proposed by Bodor [71]. In that article, it was suggested that instead of increasing the tibial shear force, a quadriceps contraction compresses the tibiofemoral joint with a fixed foot and pulls the femur distally and anteriorly. In doing so, the quadriceps would decrease the GRFy values at landing. Logerstedt et al. noted decreased quadriceps strength in National Collegiate Athletic Association athletes who continued to have self-reported knee function limitations after ACL reconstruction [72]. Rather than facilitating or causing an anterior tibial shear force, their findings support the notion that increased quadriceps strength relative to the hamstrings may provide improved force dissipation with landing. Additionally, Schmitt et al. reported that decreased quadriceps femoris strength was associated with increased vertical ground reaction force in the weaker LE (*p* < 0.001) [73].

The second predictor variable in the nGRFy regression model (FFM) had a direct influence on the dependent variable. As the FFM increased, the magnitude of the posterior GRF decreased (moved anteriorly). The greater overall muscle mass, then, has the potential to increase the potential muscular output, and thus improve the landing energy dissipation potential. Successful ACL injury prevention programs have placed an emphasis on muscular output. Programs that have focused on neurological activation alone [74,75,76] are reportedly less effective than those that address both neurological and muscular components [77,78]. The incorporation of activation and muscular output variables in our GRF equations confirms the important role of both components in landing energy dissipation.

The third predictor variable also had a direct influence on nGRFy, in that the magnitude of the posterior GRF decreased as the passive ankle dorsiflexion increased. As Fong et al. also reported [42], reduced ankle dorsiflexion decreased the knee flexion at landing. Decreased knee flexion could magnify the effect of any anterior pull of the quadriceps, and thus any shear force. This shear force could be recorded as a greater magnitude of posterior GRF [58]. Thus, decreased DPROM should increase the posterior vector of the GRFy.

### 4.3. Selection of Examined Variables

Previous research has explored a variety of methods to predict those who will succumb to an ACL injury [14,15,79]. Within any study participant pool, the frequency of ACL injury is prospectively unknown. In response to this challenge, investigators have utilized known ACL injury risk factors as a proxy for the actual injury [17,58,63,80,81,82,83,84,85,86,87,88,89]. The potential for ACL injury incidence is higher with larger sample pools. In the event that a considerably larger sample is a viable option, researchers have attempted to predict actual incidence rather that utilize ACL injury risk factors as a proxy. The identified factors with a direct impact on ACL injury risk are elevated GRF_z_ and GRF_y_ with single LE landing. These factors do not provide practical measurement methods. If a practical and low-cost method of predicting GRFs during single-leg landing were devised, then an identification strategy would be available for those at elevated risk for ACL injury.

A considerable volume of ACL injury research has identified multiple ACL injury risk factors [1,10,11,12]. The literature classifies these risk factors into intrinsic (anatomical and hormonal) and extrinsic (environmental and biomechanical) categories [1], each with varying degrees of practical control. As these injuries are common in environments where financial, equipment, and trained personnel resources are limited, we chose to explore the predictability of variables where practical and cost-effective assessment tools would identify pathways for practical and cost-effective interventions. These tests and interventions then could potentially have the greatest impact on the population at elevated risk.

Intrinsic risk factors for ACL injury such as anatomical factors also offer less practicality to testing and intervention due to the drastic or invasive measures required. Intrinsic risk factor intervention is recognized as being under less control [13], and without robust and unequivocal evidence for support [90]. The literature has detailed varying rates of ACL injury incidence in the phases of the menstrual cycle with elevated risk in both the follicular and luteal phases [91,92], in the ovulatory phase [93,94], or in the menstrual or pre-menstrual phase [95,96]. There is also evidence of no significant change in laxity over the menstrual cycle [97,98,99,100,101,102,103]. The lack of a clearly described risk profile from each sex hormone suggests the possibility that the disparity of ACL injury rates may result from other risk factor(s), with sex acting at least partially as a confounder. In our findings, it should be noted that sex was statistically significant for GRFy, but not GRFz, and did not appear in the most economical model for either dependent variable. To the authors, this suggests that the role sex plays on GRFs may be less impactful than other variables. Additionally, eliminating a sex-based hormonal impact would not be practically applied, as it is rarely controlled for at the time of injury in an active/athletic setting. In optimizing the generalizability of our findings, we wanted female participants that described the population at risk for ACL injury as closely as possible, minimizing rather than eliminating the influence of hormonal variation instead.

The implementation of non-invasive interventions to reduce ACL injury risk would be a preferred strategy for health care professionals. Previous work has explored a robust pool of potential biomechanical and neuromuscular variables amenable to testing and intervention. Individually, these result in a modest explanation of approximately 20% variability explained for ACL injury risk [42,56]. The identification of multiple extrinsic variables might allow for a greater percent of variability to be explained by the equation(s), and thus a more robust pathway to effective intervention.

Lumbopelvic [58,86], knee [63,80,81,82,83,84,85], and ankle joint angles [87,88] at initial contact (IC) and maximal joint excursion (EXC) have been utilized as predictors for ACL injury risk. Additionally, there are noted sex differences with lumbopelvic [64,65], knee [17,59,60,61,62,63], and ankle joint kinematics [64] at landing. These kinematic differences have been thought to partially explain the sex differences with ACL injury rates. Despite these findings, the ability of joint kinematics to describe the ACL injury risk is not universally accepted [64,65,104,105]. In support of this view, the Landing Error Scoring System (LESS) [106], a commonly examined evaluation of landing kinematics, was unable to prospectively predict ACL injury in high school and college athletes [14], and offered test specificity [107] below clinically acceptable levels [40]. This may be due in part to the LESS utilizing a Double Limb Landing (DLL) versus a single-limb landing (SLL). During a DLL, the body displays different landing behaviors when compared to an SLL [108]. The literature suggests that behaviors more commonly associated with ACL injury occur with a SLL than a DLL [108,109]. Thus, we believe that our selection of SLL for examination would provide a better platform to examine the ACL injury risk.

Investigators have also explored models to predict joint kinetics that identify those at elevated ACL injury risk. Myer et al. attempted to prospectively predict knee valgus moment from the body mass index, knee flexion range of motion, tibia length, knee abduction angle, and peak knee extensor moment [89]. The authors report that they were able to predict a large percent (78%) of the variance in the knee valgus moment with the predictor variables. However, one of their predictor variables required the utilization of three-dimensional motion analysis equipment synchronized to a forceplate. In spite of the robust model generated with their results, this predictive strategy is not as practically applied due to the availability and operation of the required equipment.

Sturnick et al. utilized tibiofemoral architecture to successfully retrospectively identify those who had suffered an ACL injury [79]. Unfortunately, these measures required the utilization of trained health care professionals and specialized equipment (magnetic resonance imaging). As with the prediction of knee extension moments, these requirements present considerable challenges to their applications in an active/athletic setting.

Elevated undissipated vertical [15,17] and posterior [17,110] landing energy are commonly utilized proxies for ACL injury, and were also reported to predict yjr ACL injury incidence [15]. Peak ACL load with both SLL [111] and DLL [58] occurs when peak GRFz and GRFy occur [58,111]. Thus, we chose to examine the peak GRFs as a proxy for predicting the ACL injury incidence in the current investigation.

Video analysis has suggested that ACL injury failure occurs within a very limited time window lasting from IC to as little as 0.1 s [63,112]. Observed peak GRFs are known to occur within this 0.1 s time window, while the kinematic analysis of max EXC requires considerably longer [113]. If maximal loading of the ACL occurs when peak GRFs occur as reported, the examination of ACL injury risk should capture the moment when peak GRFs occurs. Our data specified that the mean time to peak GRFz occurred at 0.060 ± 0.014 s, while the mean time to peak GRFy occurred at 0.035 ± 0.031 s, with both occurring well within the 0.1 s after IC time window. Based upon this, we believe that our data provides evidence that our collection time window strategy for the dependent variables of 0.1 s after IC captured the moment when an ACL would potentially be damaged during an SLL.

Previous work has shown that landing energy dissipation and muscular output are correlated [56]. Our findings agree as nGRFy was significantly correlated with all, and nGRFz most, of the muscular output variable values. It has also been noted that females produce decreased peak muscular output versus males when not matched for mass or maturational status [114,115]. Our findings agree with this, as there were significant correlations between sex and MK, SLTH, HipLR, and FFM. Taken together, these results raise the question of whether the apparent sex difference in the ACL injury rate may actually be from a sex difference in muscular output. This premise has been suggested by others [21]. It would behoove future investigators to examine the role of muscle activation and increased FFM to landing energy dissipation in ACL injury prevention. In the case of FFM, the authors believe this would be especially important in young female athletes, which is a population at elevated risk of ACL injury [13,116,117,118,119].

The ODS is commonly scored on a 0–3 scale when utilized in the Functional Movement Screen (FMS) [120]. Utilizing an ordinal variable in a regression equation requires an equal number of regressions iterations, as there are possible scores in that variable. The utilization of four regression iterations (0–3), then, creates an additional source of potential error. Our interest in utilizing the ODS was to determine if there was a functional limitation of fixed foot LE joint range of motion. After receiving a LEFS of greater than 71/80 and passing the manual range of motion screen, we assumed that there would be no participants with a profound degree of limitation. The ODS was therefore assessing an LE limitation on either a “3” or “2” score within the FMS framework. The utilization of a dichotomous score does not introduce as much possible error as an ordinal score with four possible scores. As the information we were seeking could be provided with a dichotomous score, utilizing this scoring provided less possible error in the regression analysis.

The amount of variance explained by the nGRFy model is considerable (48%), especially in comparison to the other ACL injury or GRF predictive models reported in the literature [42,56]. Additionally, predictive models are prone to the considerable variation in humans, often resulting in *r*^2^ or *R*^2^ values between 0.11 and 0.28 [121,122,123,124]. As the amount of variance predicting GRFs nearly doubles previous strategies, the use of the nGRFy model would allow for improved efforts to identify those at elevated ACL injury risk. Efforts to utilize such a model in this population should await the validation of the current nGRFy findings. The amount of variance explained by the nGRFz regression was statistically significant but limited to 27%. The significance of the model suggests that efforts to investigate additional unexplained variance via other practical measures would be advantageous. Unfortunately for current application purposes, the utilization of the nGRFz model explains too little of the variance to be clinically meaningful in comparison to other efforts that described GRFs [42,56].

As with all research investigations, we understand that there are limitations. Among these are limits from our research design, efforts to control error, limits within our selected measurement tools, and within our selected sample. The awareness of these limitations has allowed us to implement strategies to minimize the effects when possible.

### 4.4. Clinical Implications

Although both models were statistically significant, the nGRFz model explained only 27% of the variance, while the nGRFy model explained 48% of the variance. The range of human variability has prevented three decades of extensive investigations from perfectly describing landing behaviors. In an effort to improve injury prevention efforts, the values in the current investigation are slightly (nGRFz) to considerably above (nGRFy) previous research results to predict GRFs [42,56].

The clinical use of the nGRFz equation in the current study provided only a slightly improved predictive value (additional 1–9% the variance explained) versus the prior reported strategies. Although our findings show an improvement over previous investigative efforts, the equation still results in only a small to moderate effect size. As such, the use of the nGRFz model as reported would be of lesser value for the clinician working with healthy and active individuals. The use of other measures to explain additional variance in this model would certainly be beneficial.

If validated, the nGRFy model, utilizing only three practical clinical measures, may be a valuable tool in an effort to identify those at elevated risk of ACL injury due to elevated GRFs. The importance of our findings is considerable when compared to the predictive values of previously explored strategies. The nGRFy model explains more than double the variance (*r*^2^ = 0.22 versus adjusted *R*^2^ = 0.48) when compared to the next most effective GRF approach [56]. Improved predictive ability then should allow for the better identification of those at elevated ACL injury risk. Additionally, as the selected clinic and functional tests are often already performed as a component of an athlete’s pre-season participation activities, the calculation of nGRFy would require minimal additional time. The selection of tests also would be both practical and cost-effective for field-based sports medicine or strength and conditioning practitioners in these settings.

## 5. Conclusions

This study utilized common clinical measures and functional tests, plus sex, to predict vertical and posterior GRFs in a sample of healthy and active college-age individuals. Our findings showed that 27% of the variance in predicting vertical GRF could occur from the results of SLTH and DPROM. We also found that the results of H:Q, FFM, and DPROM could explain 48% of the variance in the posterior GRF model for our sample. In selecting GRFs as identified ACL injury risk factors, these findings suggest that, if verified, practical methods to identify individuals at elevated risk of ACL injury exist.

## Figures and Tables

**Table 1:** Analysis of demographic statistics.

	Female Mean ± SD	Male Mean ± SD	Sex Difference Significance
Age (years)	20.667 ± 1.461	21.571 ± 1.287	*p* = 0.039 *
Height (cm)	65.524 ± 1.874	70.702 ± 2.363	*p* < 0.001 *
Mass (kg)	64.190 ± 9.059	82.202 ± 7.606	*p* < 0.001 *
LEFS Score	79.524 ± 1.250	79.143 ± 1.558	*p* = 0.387
Beighton Score	2.095 ± 1.640	0.476 ± 0.750	*p* < 0.001 *
Self-reported days active in past six months	4.762 ± 1.221	4.667 ± 1.133	*p* = 0.795

* Significance *p* < 0.05.

**Table 2:** Means and standard deviations for independent and dependent variables.

Variable	Female Mean ± SD	Male Mean ± SD	Sex Difference Significance
FFM	47.487 ± 3.684 kg *	72.297 ± 5.835 kg	*p* < 0.001 *
DPROM	17.444 ± 5.015° *	13.460 ± 7.359°	*p* = 0.047 *
MK	946.761 ± 159.423 Watts	1412.310 ± 225.437 Watts	*p* < 0.001 *
SLTH	429.825 ± 42.660 cm *	539.175 ± 53.724	*p* < 0.001 *
HipLR	145.623 ± 27.041 N *	206.078 ± 34.486 N	*p* < 0.001 *
H:Q	0.828 ± 0.137	0.767 ± 0.092	*p* = 0.097
nGRFz	4.463 ± 0.896	4.061 ± 0.935	*p* = 0.163
nGRFy	−3.801 ± 0.910 FFM *	−2.816 ± 0.989 FFM	*p* = 0.002 *

* Significance *p* < 0.05. Fat Free Mass (FFM), Ankle dorsiflexion passive range of motion (DPROM), Margaria–Kalamen test (MK), Single Leg Triple Hop (SLTH), Hip lateral rotator muscles peak force (HipLR), Hamstrings to quadriceps peak force ratio (H:Q), Fat Free Mass normalized Vertical (nGRFz) and Posterior ground reaction forces (nGRFy).

**Table 3:** Correlation matrix for nGRFz and the independent variables.

	nGRFz	SLTH	MK	DPROM	H:Q	FFM	HipLR
nGRFz	*r* = 1.00	*r* = −0.399 *	*r* = −0.336 *	*r* = −0.335 *	*r* = 0.309 *	*r* = −0.258	*r* = −0.186
		*p* = 0.009	*p* = 0.030	*p* = 0.030	*p* = 0.047	*p* = 0.098	*p* = 0.238
SLTH		*r* = 1.00	*r* = 0.752 *	*r* = −0.126	*r* = −0.407 *	*r* = 0.694 *	*r* = 0.511 *
			*p* < 0.001	*p* = 0.427	*p* = 0.008	*p* < 0.001	*p* < 0.001
MK			*r* = 1.00	*r* = −0.204	*r* = −0.417 *	*r* = 0.753 *	*r* = 0.657 *
				*p* = 0.195	*p* = 0.006	*p* < 0.001	*p* < 0.001
DPROM				*r* = 1.00	*r* = −0.221	*r* = −0.295	*r* = −0.302
					*p* = 0.159	*p* = 0.058	*p* = 0.052
H:Q					*r* = 1.00	*r* = −0.285	*r* = −0.316 *
						*p* = 0.068	*p* = 0.042
FFM						*r* = 1.00	*r* = 0.711 *
							*p* < 0.001
HipLR							*r* = 1.00

* Significance *p* < 0.05.

**Table 4:** Correlation matrix for nGRFy and the independent variables.

	nGRFy	H:Q	FFM	MK	SLTH	HipLR	DPROM
nGRFy	*r* = 1.00	*r* = −0.530 *	*r* = 0.528 *	*r* = 0.521 *	*r* = 0.459 *	*r* = 0.400 *	*r* = 0.228
		*p* < 0.001	*p* < 0.001	*p* < 0.001	*p* = 0.002	*p* = 0.009	*p* = 0.147

* Significance *p* < 0.05.

**Table 5:** Point bi-serial correlations for continuous variables and dichotomous variables.

	nGRFz	nGRFy	FFM	DPROM	MK	SLTH	H:Q	HipLR
Sex	r_pb_ = −0.219 *p* = 0.163	r_pb_ = 0.472 * p = 0.002	r_pb_ = 0.934 * p < 0.001	r_pb_ = −0.308 * p = 0.047	r_pb_ = 0.774 * p < 0.001	r_pb_ = 0.756 * p < 0.001	r_pb_ = 0.259 *p* = 0.097	r_pb_ = 0.707 * p < 0.001
ODS	r_pb_ = −0.267 *p* = 0.087	r_pb_ = 0.095 *p* = 0.548	r_pb_ = −0.125 *p* = 0.429	r_pb_ = 0.473 * p = 0.002	r_pb_ = 0.075 *p* = 0.635	r_pb_ = 0.095 *p* = 0.549	r_pb_ = −0.064 *p* = 0.688	r_pb_ = −0.095 *p* = 0.549

* Significance *p* < 0.05.

**Table 6:** Pearson chi-squared analysis for overhead deep squat (ODS) and sex.

		ODS–Fail	ODS–Pass
Female	Number	7	14
	Percent	0.333	0.666
Male	Number	10	11
	Percent	0.476	0.524

**Table 7:** Regression table for stepwise multiple linear regression analysis for nGRFz from predictor variables.

Variable	Coefficient	Error	T	*p*	Model Adjusted R^2^	Model *p*
Constant	7.868	0.916	8.589	<0.001	0.274	0.001
SLTH	−0.006	0.002	−3.340	0.002		
DPROM	−0.055	0.019	−2.918	0.006		

**Table 8:** Regression Table for Stepwise Multiple Linear Regression Analysis for nGRFy from Predictor Variables.

Variable	Coefficient	Error	T	*p*	Model Adjusted R^2^	Model *p*
Constant	−4.394	1.373	−3.200	0.003	0.476	<0.001
H:Q	−2.579	1.070	−2.410	0.021		
FFM	0.041	0.010	4.197	<0.001		
DPROM	0.041	0.020	2.060	0.046		
